# Pattern of presentation of cervical carcinoma at Nuclear Institute of Medicine and Radiotherapy, Pakistan

**Published:** 2013

**Authors:** Nousheen Aziz, Sajida Yousfani

**Affiliations:** 1 Dr. Nousheen Aziz, MS, Senior Registrar, Department of Gynaecology & Obstetrics, Liaquat University of Medical & Health Sciences, Jamshoro, Sindh, Pakistan.; 2 Dr. Sajida Yousfani, FCPS, Associate Professor, Department of Gynaecology & Obstetrics, Liaquat University of Medical & Health Sciences, Jamshoro, Sindh, Pakistan.

**Keywords:** Cervical cancer, Frequency, Stage at diagnosis

## Abstract

***Objective: ***To find the pattern of presentation of cervical carcinoma as seen at Nuclear Institute of Medicine and Radiotherapy, Pakistan.

***Methodology: ***This retrospective descriptive study was conducted at the Nuclear Institute of Medicine and Radiotherapy (NIMRA) for a period of one year from January 1^st^ to December 31^st^, 2009. The clinical records of all patients diagnosed with carcinoma of the cervix were reviewed with regard to sociodemographic and reproductive parameters. Frequency of cervix and stage of disease at time of presentation were recorded. The data was collected on pre-designed proforma and analysed using SPSS Version 16 statistical package.

***Results*** *:* There were a total of 56 cases (33.53%) of carcinoma of cervix who presented at NIMRA during the study period. The mean age was 51 years. Irregular vaginal bleeding, foul smelling vaginal discharge and post coital bleeding were the most common symptoms. Squamous cell carcinoma corresponds to 52 (92.85%) and adeno carcinoma to 4 (7.14%) cases. Only 8(14.28%) cases were in Stage I, while 20(35.71%), 22(39.28%) were in Stages II and Stage III respectively whilst 6(10.71%) cases were in advanced stage (1V).

***Conclusion:*** Squamous cell carcinoma accounted for 92.85% of cases with mean age of 51 years, most patients 74.98% presented in stage II, III and IV. Diagnosis at advanced stage needs implementation of large scale educational and screening programme on national level to saves the lives of Pakistani women.

## INTRODUCTION

Cervical cancer represents a major global public health problem for women of all ages. Carcinoma of cervix accounting for about 12% of all cancer cases globally, is a preventable disease that continues to occur as the second most common cancer among women throughout the world.^1^

There is a unique geographical distribution which has been published by a group called Globocan to estimate the amount of cases and incidence of cervical cancer. The uniqueness is that cervical cancer incidence is highly concentrated in the southern part of the hemisphere. The highest concentration is in central South America which constitutes about 71,000 cases a year, and sub-Saharan Africa which constitutes 78,000 a year, followed by India and Southeast Asia, which also has 260,000 cases occurring in a year. The lowest incidence for this cancer occurs in North America, Europe, and Australia.^2^

Globally 493,000 new cases of cervical cancers are diagnosed annually and it kills approximately 274,000/ women per year.^3^ Developing countries carry two thirds of the burden of the disease as 85% of deaths occur in these resource poor countries.^4^ The burden of cancer in low and medium income countries (LMIC) is expected to increase in the next decades.^5^

According to American Cancer Society, it was estimated that in the US 11,070 new cases and 3870 deaths due to cervical cancer was reported in 2008.^6^ In India, 13200 new cases were diagnosed and 74000 deaths occur annually.^7^

Cervical cancer deaths are projected to rise by almost 25% over the next ten years.^8^ Age-specific cervical cancer mortality rates per 100,000 women again show us another disparity between less developed countries and developed countries. If we look at the age between 45 and 54, almost five times more women were dying in the less-developed countries compared to the developed countries.^2^

The discrepancy between developed and developing countries on cervical cancer incidence and mortality is also paralleled by differences in educational levels, knowledge of cervical cancer and its prevention.^9^

In Pakistan, the exact incidence and prevalence of carcinoma of cervix is not known. Current scenario regarding screening is that conventional cytology is offering sporadically to women in selected urban areas attending health services for other reasons not as routine screening in asymptomatic women so uptake of screening is currently suboptimal. It was reported in one study that only 5% percent of women in Pakistan were aware of screening and only 2.6% of women actually had PAP smear done once.^10^ Moreover, screening is not available in most parts of the country.

Carcinoma of cervix is a relatively neglected disease in terms of advocacy, screening, prevention from professional & public health organisations at least in our part of the world.

This study was carried out to determine the pattern of presentation of carcinoma of the cervix at the Nuclear Institute of Medicine And Radiotherapy with reference to the stage at presentation in order to institute measures for early diagnosis and presentation. This will lead to a reduction in mortality from carcinoma of the cervix.

## METHODOLOGY

This retrospective descriptive study was conducted at Nuclear Institute of Medicine and Radiotherapy (NIMRA) for a period of one year from January 1^st^ to December 31^st^, 2009. All patients diagnosed histopathologically and registered as carcinoma of cervix were included in the study. The clinical records of all patients were reviewed with regard to sociodemographic and reproductive parameters including age, parity, pre or postmenopausal status. Frequency of carcinoma cervix and stage of disease at time of presentation were recorded. The data was collected on pre-designed proforma and analysed by using SPSS Version 16 statistical package. Descriptive statistics were used for demographic data and summarized as means with standard deviation or frequency with percentage.

## RESULTS

There were a total of 167 patients who presented with different female genital tract neoplasia at Nimra Jamshoro during the study period, which included 56 cases (33.53%) of carcinoma of the cervix.

**Table-I:** Demographic characteristics and symptoms

*Characteristic*	*No & % *
Age of Patients	
30 <30 years	3(5.35%)
31-40 years	6(10.71%)
41-50 years	20(35.71%)
50-60 years	14(25.00%)
61-70 years	11(19.64%)
>70 years	2(3.57%)
Parity	
P 1-4	11(19.64%)
P5-7	23(41.07%)
P8-10	15(26.78%)
P>10	7(12.5%)
Symptoms	
Postmenopausal bleeding	17(30.35%)
Irregular Vaginal bleeding	27(48.21%)
Post coital bleeding	3(5.35%)
Vaginal Discharge	25(44.64%)
Pain in lower abdomen	16(28.57%)
Any other	17(30.35%)
Menopausal status	
Pre-menopause	31(55.35%)
menopause	25(44.65%)
Socio Economic status	
<5000Rs	98(96.42%)
>5000Rs	2(3.57%)

The results are expressed in numbers and percentage

**Fig.1:**
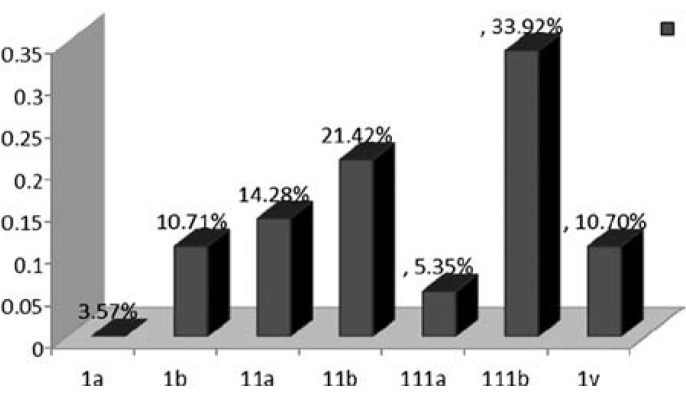
Stages of carcinoma of cervix. The results are expressed in percentage

Age range of women was between 29 and 73 years with mean age 51 years. Three women (5.35%) were aged less than 30 years, six women (10.71%) were aged between 31-40 years, 20 women (35.71%) between 41-50 years, 14 women (25.00%) between 50-60 years, 11 women (19.64%) were between 61-70 years, while 2 women (3.57%) presented after 70 years of life. As shown in Table-I, regarding parity, 11 women (19.64%) were para 1-4, 23 women (41.07%) were Para 5-7, 15(26.78%) and 7 women (12.5%) were para 8-10 and >10 respectively.

The most common symptoms in present study were Irregular Vaginal bleeding 27(48.21%), Vaginal Discharge 25(44.64%), and Postmenopausal bleeding in 17(30.35%) of women with carcinoma of cervix. Out of 56 cases of carcinoma of cervix 31 women (55.35%) and 25 women (44.65%) were in Pre-menopausal and post menopausal states respectively. Ninety-eight women (96.42%) belonged to low socio-economic class.

The most frequent histological type was Squamous cell carcinoma corresponding to 92.85% and Adenocarcinoma to 7.14% of the cases respectively. There were only 8 cases (14.28%) in Stage I, while 20(35.71%), 22(39.28%) cases were in Stage II and Stage III respectively. Six cases (10.71%) were in advanced stage (1V) as shown in Fig.1.

## DISCUSSION

The present study was carried out to analyze the patients with carcinoma of cervix for stage of disease at time of presentation, so that measures are taken for prevention and early diagnosis to reduce mortality. At NIMRA 56 (33%) of cases of carcinoma of cervix who were detected during one year make it 1^st^ common cancer of female genital tract and second commonest malignancy that affects women after breast cancer. The demographic profile of the patients with cervical cancer in our study was as middle aged women of reproductive age presenting late with advanced stage of the disease and of lower socioeconomic status. The findings are compatible with national and international studies.^11^^,^^12^

The age range of patients at time of presentation in this study was between 29 and 73 years with mean age 51 years comparable to the study conducted by Kaku who reported the mean age of 56 years with age range of 33-82 years.^13^

In our study 51.77% of the cases were seen in women below 50 years, comparable to results in the US where more than half (58%) and 45% of the cases in Mumbai were seen in women below 50 years. In our study, there is gradual increase in carcinoma of cervix from fourth up till the seventh decade followed by an actual decrease in risk, while peak incidence was observed in the 41-50 years age group. Similar results were found in a similar study except that peak incidence was observed in the age group 60-64 years.^14^ The determination of age trends at presentation of cervical carcinoma are important as this helps in the identification of the target group for the implementation of cervical screening proramme.

The morphological categorization is also important for management of cancer of the cervix. In our study Squamous cell carcinoma and adenocarcinoma were found in 92.85%, 7.14% of cases. In the entire Asian region, squamous cell carcinoma of the cervix has been reported as a major problem,^15^ while western population have recently documented a morphological shift with a rising adenocarcinoma component of cancer of cervix (10%).^16^

The incidence and mortality levels differ significantly within every stage.^17^ The clinical stage at the time of diagnosis was very important as treatment of cancers in the earlier stages produce better results,^18^ as prognosis depends on it^19^ and does save lives.

In our study majority (74.98%) of patients present in advanced stage compared with the results from Lahore where 54.92% presented in advanced stage that is II and III.^20^ It seems that cancer of cervix is quite prevalent in Pakistan with increasing incidence. Data from different hospital provides sound evidence for increasing trends of cervical cancer^21^ however for exact incidence National cancer registry is necessary.

The high incidence of this cancer and its detection at advanced stage, and increasing cost of management day by day with poor survival rate and its burden born by patient, and their family or society in large are all factors which call for urgent measures to prevent these death by large scale public health educational and screening programmes.

Vaccination of young women against HPV Human papilloma viruses has been shown to be very efficacious in preventing the development of moderate to severe cervical precancerous lesions associated with HPV 16-18.^22^

 Prophylactic HPV vaccination should be included in national immunization programme offered to the prepubertal girl. To achieve this target there is a need to reformulate and implement HPV vaccination programme in developing countries like Pakistan with collaboration of WHO.

Due to lack of facilities and financial resources, adequate coverage of entire female population by cytology based screening programs is not at all feasible in developing countries. Down staging represents a potentially important approach for cancer control and priority area for future research. Alternative strategies including clinical down staging of cancer cervix, through a single life time screening appears to be more feasible and affordable mode to control of carcinoma of cervix in developing countries like Pakistan.

Keeping the data in perspective of our study that is that peak incidence was found at earlier age as compare to other studies, the once in a lifetime method of cervical cancer screening should be applied to the 30-40 years age group using HPV DNA testing. It will help to pick up and treat cases earlier to prevent mortality. Once-in-a-lifetime screening between the ages of 35 and 40 can reduce lifetime cervical cancer risk by 25% to 35%.^23^

While the primary prevention by prophylactic HPV vaccination in prepubertal women provides the hope for the future, the current means of secondary prevention of cervical cancer screening with HPV DNA testing for older women will help to reduce the incidence of carcinoma of cervix.

To maximize participation of women in screening and treatment and to improve cost-effectiveness and efficacy, it has proposed that along with single or a twice in a life time screening targeting women aged 30-49, 30-59 using HPV DNA testing as a primary screening test followed by cytology tri-age or VIA triage, and use of single visit approach that entails treatment of VIA or HPV positive women by cryotherapy, with no evidence of invasive cancer in same sitting in low resource setting.^5^ Large trials are needed before application of such type of progrmmes.

This study has several limitations being retrospective analysis that relied heavily on medical records so prospective studies are required with large sample size. It is very necessary to update the national cancer control strategies, the policies influencing HPV vaccination and screening need to be reassessed at multiple levels to achieve more effective implementation. Incidents of cervical cancer in Pakistan are on the rise but through effective screening and vaccination we can successfully fight this deadly disease.

Finding of this study will help in taking effective measures for prevention and early diagnosis of cervical cancer to reduce mortality in our country.

## CONCLUSION

Diagnosis of cervical cancer at advanced stage II, III and IV(85.70%) needs implementation of large scale educational and screening programme on national level to saves the lives of Pakistani women.
